# Knowledge, Attitude, and Practices Related to Vitamin D, Zinc, and Ferritin Deficiencies and the Associated Risk of Hair Loss in Jazan, Saudi Arabia

**DOI:** 10.7759/cureus.48731

**Published:** 2023-11-13

**Authors:** Ahmad Assiri, Abdulaziz Rajhi, Abdulrahman Sudi, Atheer Akoor, Shumokh Juraybi, Naif K Mahzara, Yumna Abutalib, Abdulaziz M Kariri, Tahani Altubayqi, Anas A Madkhali

**Affiliations:** 1 Dermatology, Faculty of Medicine, Jazan University, Jazan, SAU; 2 Medicine, Faculty of Medicine, Jazan University, Jazan, SAU

**Keywords:** jazan, supplements, sun exposure, practices, knowledge, hair loss, deficiencies, ferritin, zinc, vitamin d

## Abstract

Background: Vitamin D, zinc, and ferritin deficiencies are prevalent worldwide and linked to various health outcomes, including hair loss. This study aimed to assess the knowledge, attitude, and practices related to these deficiencies among citizens in Jazan, Saudi Arabia, and their perceptions regarding the relationship between these deficiencies and hair loss.

Methods: A cross-sectional study using a self-administered questionnaire was conducted between May 2023 and July 2023 among 985 participants, predominantly young adults aged between 18 and 25 years (58.9%, n = 580), females (56.2%, n = 554), single individuals (65.2%, n = 642), and Saudi citizens (98.2%, n = 967). Information on participants' demographics, knowledge, and practices related to vitamin D, zinc, and ferritin deficiencies was collected through Google Forms (Alphabet Inc., Mountain View, CA, USA). Knowledge levels were categorized as high or low based on responses.

Results: Almost all participants (95.2%, n = 938) had heard about vitamin D, zinc, and ferritin deficiencies and their risk of hair loss, and 554 (56.2%) participants had high knowledge, whereas 431 (43.8%) had low knowledge. The majority used the Internet to get information (37.1%, n = 365), identified sunlight as a source of these vitamins and minerals (91.7%, n = 903), and believed in their association with hair loss (74.0%, n = 729). Only about a third of participants reported daily sun exposure of 34.9% (n = 344) and vitamin supplement use of 35.4% (n = 349). Regression analysis revealed gender, awareness of vitamins, and vitamin supplement intake as significant factors related to hair loss (p < 0.001).

Conclusions: The findings underscore the need for health education to improve sun exposure and supplement use practices, which could potentially reduce the prevalence of these deficiencies and associated hair loss. Future research should consider exploring other factors influencing hair loss and extending the study to different demographic groups.

## Introduction

Vitamin D, zinc, and ferritin are vital minerals crucial to overall health and well-being [1,2]. If these minerals are deficient, they can significantly negatively affect various bodily functions, including the health of our hair [3,4]. While genetic factors and hormonal imbalances contribute to hair loss, recent studies have highlighted the role of deficiencies in overall well-being [5,6].

Healthy-looking hair is not solely the result of good hair care practices; it also indicates excellent general health [3,7]. Most healthy individuals maintain a nutrient-rich diet. However, many people lack access to proper nutrition or have medical conditions that make them prone to nutritional deficiencies [3]. These deficiencies often manifest as changes in the scalp and body hair, such as alterations in hair color, weakening, and even hair loss [8,9]. Various conditions, including malnutrition, chronic illnesses, and advanced age, can contribute to these changes [8,9].

Identifying specific populations that may be at higher risk for vitamin deficiencies is an important first step in detecting and addressing these deficiencies on a broader scale. Variations in hair texture can serve as valuable indicators of underlying vitamin deficiencies [9,10]. Consequently, people often inquire about vitamin supplementation as a potential means to prevent or treat dermatological conditions, particularly hair loss [10,11].

Despite growing awareness of the link between deficiencies and hair loss, some key gaps remain. Specifically, knowledge, attitudes, and practices regarding these deficiencies are not well understood. This lack of understanding is especially true among the general population in the Jazan region of Saudi Arabia. This study aims to assess the knowledge, attitudes, and practices of the general population in Jazan towards vitamin D, zinc, and ferritin deficiencies and their potential risk for hair loss. The findings from this research will raise awareness and provide valuable insights or concrete evidence that decision-makers can use to implement effective interventions. These interventions could include monitoring systems and educational programs to address the risks of hair loss associated with these mineral deficiencies. This can help alleviate a concern that significantly affects individuals' self-esteem and quality of life.

## Materials and methods

Study design and settings

This study employed a cross-sectional design using online questionnaires among the general population in Jazan. The study aimed to understand the features and behaviors of the general population concerning vitamin D, zinc, and ferritin deficiencies and their potential impact on hair loss.

Study population and inclusion/exclusion criteria

The target population for this study included adults from the general population of Jazan. Participants were recruited through online platforms, ensuring a diverse representation of individuals residing in Jazan. Individuals who resided in the Jazan region, especially those aged 18 and above, and were willing to participate were included. Individuals who refused to participate, those under 18, or those who resided outside the Jazan region were excluded.

Sampling technique

Participants were recruited using convenience sampling. This technique involves selecting individuals who are readily available and willing to participate. Participants were recruited through various online platforms, such as social media groups, online forums, and community networks, to ensure a broad representation of the general population in Jazan. All recruited participants were provided with an online questionnaire link to complete at their convenience.

Sample size

The sample size for this study was calculated at 385 using the following formula for probability sampling: 𝒏 = 𝒛2𝟏−𝜶 𝑷(𝟏−𝑷)/ 𝒅𝟐, where n is the sample size, z is a standard normal distribution (1.96 to a confidence level of 95%), p is the anticipated population proportion (50%) for the maximum sample size, and ‏d = error not more than 0.05. Considering a 10% non-response rate, the required sample size was initially estimated to be 385.

However, we were able to recruit a substantially larger sample of 985 participants using social media-based advertising and outreach through community networks. The opportunity to include a larger sample provided greater statistical power for our analyses, particularly in evaluating differences between sociodemographic subgroups. Additionally, the increased sample size improves the overall generalizability of the results to the general population in Jazan. It also accounts for any incomplete or missing survey responses. Therefore, the final study sample consisted of 985 participants, much larger than our initial target.

Data collection process

Data were collected between May 2023 and July 2023 via a self-administered online questionnaire. The data collection instrument was designed and modified after an extensive review of past studies [4,12]. The data collection tool used in this study is a structured questionnaire. The survey was split into two primary parts: (1) general information and (2) knowledge and practice about vitamin D, zinc, ferritin deficiency, and the risk of hair loss.

The general information section collected sociodemographic data about the participants, including gender, age, residence, nationality, and marital status. The second section assessed the participants' knowledge and practices concerning vitamin deficiency and its potential risk of hair loss. It included questions about their source of information, understanding of the sources and benefits of these minerals, causes of their deficiency, and the potential health impacts of such deficiencies, with a particular focus on hair loss. It also included questions about their daily activities, sun exposure habits, diet, and use of supplements. The questionnaire used a combination of multiple-choice and yes-or-no questions to collect data. Participants were asked to select the most appropriate response that represented their situation or viewpoint.

Pilot study

A pilot study tested whether the questionnaire language was clear and understandable. Each participant was asked to read and agree to an online consent form before the start of data collection. The final poll questionnaire was administered in Arabic.

Statistical analysis

Data management and analysis were performed on SPSS Statistics version 26 (IBM Corp., Armonk, NY, USA). Categorical variables are represented as frequencies and percentages. For the knowledge questions, each correct response was scored 1 point, and the incorrect response was 0. A total knowledge score was calculated for each participant. Bivariate analysis was conducted using chi-square tests to assess the relationship between knowledge, attitudes, practices, and sociodemographic factors. Logistic regression analyses were conducted to explore the relationship between these factors and the risk of hair loss. A p-value of 0.05 indicated a statistically significant difference between the variables. Tables and figures were used to express the results.

Ethical considerations

Ethical approval was obtained from the Scientific Research Ethics Committee of Jazan University (approval no. REC-44/11/711). Additionally, each participant received an electronic copy of a letter of information posted online requesting their electronic permission. It was included as a preliminary cover page ahead of the online questionnaire. Respect for the dignity of the research participants was prioritized. All participants had the right to withdraw from the study at any time. All participant data was secured with high confidentiality.

## Results

There were 985 participants in the study. Most participants were young adults between 18 and 25 years old (58.9%, n = 580). More than half of the participants were female (56.2%, n = 554). Most participants were single (65.2%, n = 642) and Saudi citizens (98.2%, n = 967). The participant demographics showed that more people live in rural areas compared to urban areas, with 59.1% (n = 582) residing in villages and 40.9% (n = 403) in cities (Table 1).

**Table 1:** Sociodemographic characteristics of participants

Variable	Category	Frequency	Percentage
Age	26 to 35	122	12.4
	36 to 45	169	17.2
	18 to 25	580	58.9
	46 to 55	101	10.3
	>55	13	1.3
Gender	Male	431	43.8
	Female	554	56.2
Marital status	Widower	5	0.5
	Single	642	65.2
	Married	328	33.3
	Divorced	10	1.0
Nationality	Saudi	967	98.2
	Non-Saudi	18	1.8
Residence	City	403	40.9
	Village	582	59.1

Table 2 summarizes the assessment of knowledge and practice toward vitamin D, zinc, and ferritin deficiencies among the study participants. The majority (95.2%, n = 938) had heard about vitamin D, zinc, and ferritin before, with the Internet being the primary source of information (37.1%, n = 365). Most participants correctly identified sunlight as a source of these vitamins and minerals (91.7%, n = 903). Regarding the benefits, 33.4% (n = 329) knew about the reduced heart and vascular diseases, while 15.9% (n = 157) did not. The main causes of deficiency identified were lack of exposure to sunlight (45.8%, n = 451) and excessive use of sunscreen (17.7%, n = 174). Osteoporosis (8.6%, n = 85) and depression (40.8%, n = 402) were recognized as outcomes of the deficiencies. Most participants (74.0%, n = 729) believed a relationship between deficiencies and hair loss existed. Over half (55.2%, n = 544) considered themselves to have an average physical activity level. Only 34.9% (n = 344) had daily sun exposure, mostly less than 20 minutes (71.6%, n = 705). While 41.5% (n = 409) never used sunblock, 53.4% (n = 526) usually ate foods containing vitamins and minerals. Supplement use was reported by 35.4% (n = 349).

**Table 2:** Assessment of knowledge and practice toward vitamin D, zinc, and ferritin deficiencies

Questions	Category	Frequency	Percentage
Have you heard about vitamin D, zinc, and ferritin before?	Yes	938	95.2
	No	47	4.8
What is your source of information about vitamin D, zinc, and ferritin?	Physician	213	21.6
	The media	105	10.7
	Internet	365	37.1
	Relatives and friends	151	15.3
	I have not heard about it	37	3.8
	Other	114	11.6
What are the sources of vitamin D, zinc, and ferritin?	Sunlight	903	91.7
	Fish	25	2.5
	Eggs	1	0.1
	Water	1	0.1
	Apples	5	0.5
	I don't know	50	5.1
What are the benefits of vitamin D, zinc, and ferritin?	Strengthen the bones	211	21.4
	Stop the bleeding	20	2.0
	Memory improvement	24	2.4
	Calcium level regulation	195	19.8
	Reduce heart and vascular diseases	329	33.4
	Treat irritable bowel syndrome	20	2.0
	Treat stomach cancer	14	1.4
	Reducing muscle pain	15	1.5
	I don't know	157	15.9
What are the causes of deficiency in vitamin D, zinc, and ferritin?	Diabetes	146	14.8
	Lack of exposure to sunlight	451	45.8
	An unhealthy diet	45	4.6
	Thyroid diseases	152	15.4
	Hypertension	6	.6
	Excessive use of sunscreen products	174	17.7
	Wear heavy clothes	6	.6
	I do not know	5	0.5
Vitamin D, zinc, and ferritin deficiency may lead to?	Obese	143	14.5
	Depression	402	40.8
	Hair loss	195	19.8
	Osteoporosis	85	8.6
	Acne	4	.4
	Increased chance of cancer	5	.5
	Eczema	145	14.7
	Gastric ulcer	3	.3
	Blindness	3	.3
Is there a relationship between vitamin D, zinc, ferritin deficiency, and hair loss?	Yes	729	74.0
	No	21	2.1
	I don't know	235	23.9
Compared to your peers of the same age, do you think you are more active, less active, or average?	Less active	276	28.0
	Average	544	55.2
	More active	165	16.8
Are you exposed to sunlight daily?	Yes	344	34.9
	No	209	21.2
	Sometimes	432	43.9
What is the duration of your daily sun exposure?	Less than 20 minutes	705	71.6
	More than 20 minutes	280	28.4
Do you use sunblock products?	Usually	181	18.4
	Sometimes	235	23.9
	Never	409	41.5
	Rarely	160	16.2
Do you usually eat foods that contain vitamin D, zinc, and ferritin (fish, egg yolks, milk, cheese, yogurt)?	Usually	526	53.4
	Sometimes	392	39.8
	Rarely	67	6.8
Do you take vitamin D, zinc, and ferritin supplements?	Yes	349	35.4
	No	636	64.6

**Figure 1:**
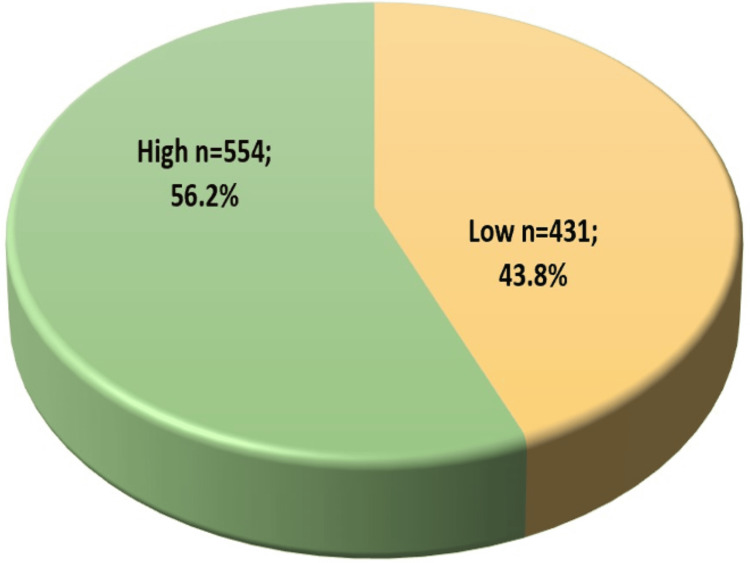
Total knowledge of participants regarding vitamin D, zinc, and ferritin deficiencies

Figure 1 indicates that a larger proportion of participants, i.e., 554 (56.2%), have high knowledge, whereas 431 (43.8%) have low knowledge about vitamin D, zinc, and ferritin deficiencies and the associated risk of hair loss.

As shown in Table 3, participants who had previously heard about vitamin D, zinc, and ferritin had significantly higher knowledge than those who had not (p = 0.004). While there were no statistically significant differences based on age, gender, marital status, nationality, or residence, a higher percentage of older (46 to 55 years) and married participants had high knowledge levels. The primary source of information for participants with high knowledge was physicians (59.6%, n = 127), followed by the Internet (55.3%, n = 202) and relatives and friends (58.9%, n = 89). Of those aged between 26 and 35, 44.3% (n = 54) had low knowledge, while 55.7% (n = 68) had high knowledge. For those aged between 36 and 45, 45.0% (n = 76) had low knowledge, and 55.0% (n = 93) had high knowledge. Among those aged from 18 to 25, 45.3% (n = 263) had low knowledge, and 54.7% (n = 317) had high knowledge. For those aged between 46 and 55, 30.7% (n = 31) had low knowledge, while 69.3% (n = 70) had high knowledge. Of those over 55, 53.8% (n = 7) had low knowledge, and 46.2% (n = 6) had high knowledge.

**Table 3:** Assessment of the association between total knowledge and demographic variables P: Pearson's chi-squared test X2, *p < 0.05 (significant)

Variable	Category	Low knowledge N (%)	High knowledge N (%)	P-value
Age	26 to 35	54 (44.3)	68 (55.7)	0.083
	36 to 45	76 (45.0)	93 (55.0)	
	18 to 25	263 (45.3)	317 (54.7)	
	46 to 55	31 (30.7)	70 (69.3)	
	>55	7 (53.8)	6 (46.2)	
Gender	Male	203 (47.1)	228 (52.9)	0.062
	Female	228 (41.2)	326 (58.8)	
Marital status	Widower	3 (60.0)	2 (40.0)	0.128
	Single	294 (45.8)	348 (54.2)	
	Married	128 (39.0)	200 (61.0)	
	Divorce	6 (60.0)	4 (40.0)	
Nationality	Saudi	422 (43.6)	545 (56.4)	0.590
	Non-Saudi	9 (50.0)	9 (50.0)	
Living	City	178 (44.2)	225 (55.8)	0.828
	Village	253 (43.5)	329 (56.5)	
Have you heard about vitamin D, zinc, and ferritin before?	Yes	401 (42.8)	537 (57.2)	0.004*
	No	30 (63.8)	17 (36.2)	
What is your source of information about vitamin D, zinc, and ferritin?	Physician	86 (40.4)	127 (59.6)	0.068
	The media	44 (41.9)	61 (58.1)	
	Internet	163 (44.7)	202 (55.3)	
	Relatives and friends	62 (41.1)	89 (58.9)	
	I have not heard about it	25 (67.6)	12 (32.4)	
	Other	51 (44.7)	63 (55.3)	

Of the male participants, 47.1% (n = 203) had low knowledge, while 52.9% (n = 228) had high knowledge. For females, 41.2% (n = 228) had low knowledge, and 58.8% (n = 326) had high knowledge. Of those widowed, 60.0% (n = 3) had low knowledge, and 40.0% (n = 2) had high knowledge. For single participants, 45.8% (n = 294) had low knowledge, while 54.2% (n = 348) had high knowledge. Among married participants, 39.0% (n = 128) had low knowledge, and 61.0% (n = 200) had high knowledge. Of those divorced, 60.0% (n = 6) had low knowledge, and 40.0% (n = 4) had high knowledge. Participants who had previously heard about vitamin D, zinc, and ferritin had significantly higher knowledge than those who had not (p = 0.004). While there were no statistically significant differences based on age, gender, marital status, nationality, or residence, a higher percentage of older (46 to 55 years) and married participants had high knowledge levels.

Table 4 shows the distribution of responses by age group for each practice question. Physical activity levels were similar across age groups, with around half of all participants reporting average activity levels and no significant differences observed (p = 0.566). Of those aged between 18 and 25 years, 29.1% (n = 169) reported being less active, 54.3% (n = 315) reported average activity, and 16.6% (n = 96) reported being more active. For ages 26 to 35, 29.5% (n = 36) were less active, 49.2% (n = 60) were average, and 21.3% (n = 26) were more active. Among ages 36 to 45, 27.2% (n = 46) were less active, 58.0% (n = 98) were average, and 14.8% (n = 25) were more active. For ages 46 to 55, 22.8% (n = 23) were less active, 62.4% (n = 63) were average, and 14.8% (n = 15) were more active. Of those over 55 years old, 15.4% (n = 2) were less active, 61.5% (n = 8) were average, and 23.1% (n = 3) were more active.

**Table 4:** Assessment of the differences in practices with age P: Pearson's chi-squared test X2, *p < 0.05 (significant)

Practices	Category	Age group/n (%)					P-value
		18 to 25	26 to 35	36 to 45	46 to 55	>55	
Compared to your peers of the same age, do you think you are more active, less active, or average?	Less active	169 (29.1)	36 (29.5)	46 (27.2)	23 (22.8)	2 (15.4)	0.566
	Averagely active	315 (54.3)	60 (49.2)	98 (58.0)	63 (62.4)	8 (61.5)	
	More active	96 (16.6)	26 (21.3)	25 (14.8)	15 (14.8)	3 (23.1)	
Are you exposed to sunlight daily?	Yes	193 (33.3)	46 (37.7)	55 (32.5)	43 (42.6)	7 (53.8)	0.186
	No	130 (22.4)	30 (24.6)	33 (19.5)	16 (15.8)	0 (0.0)	
	Sometimes	257 (44.3)	46 (37.7)	81 (47.9)	42 (41.6)	6 (46.2)	
What is the duration of your daily sun exposure?	< 20 minutes	405 (69.8)	87 (71.3)	135 (79.9)	71 (70.3)	7 (53.8)	0.069
	> 20 minutes	175 (30.2)	35 (28.7)	34 (20.1)	30 (29.7)	6 (46.2)	
Do you use sunblock products?	Usually	141 (24.3)	22 (18.0)	11 (6.5)	7 (6.9)	0 (0.0)	0.001*
	Sometimes	129 (22.2)	26 (21.3)	53 (31.4)	22 (21.8)	5 (38.5)	
	Never	214 (36.9)	56 (45.9)	79 (46.7)	54 (53.5)	6 (46.2)	
	Rarely	96 (16.6)	18 (14.8)	26 (15.4)	18 (17.8)	2 (15.4)	
Do you usually eat foods that contain vitamin D, zinc, and ferritin (fish, egg yolks, milk, cheese, yogurt)?	Usually	303 (52.2)	65 (53.3)	94 (55.6)	56 (55.4)	8 (61.5)	0.471
	Sometimes	234 (40.3)	45 (36.9)	65 (38.5)	43 (42.6)	5 (38.5)	
	Rarely	43 (7.4)	12 (9.8)	10 (5.9)	2 (2.0)	0 (0.0)	
Do you take vitamin D, zinc, and ferritin supplements?	Yes	177 (30.5)	51 (41.8)	65 (38.5)	49 (48.5)	7 (53.8)	0.001*
	No	403 (69.5)	71 (58.2)	104 (61.5)	52 (51.5)	6 (46.2)	

Daily sun exposure and duration of exposure also did not differ significantly by age (p = 0.186 and p = 0.069, respectively). Of those aged between 18 and 25 years, 33.3% (n = 193) had daily sun exposure. This was 37.7% (n = 46) for ages 26 to 35, 32.5% (n = 55) for ages 36 to 45, 42.6% (n = 43) for ages 46 to 55, and 53.8% (n = 7) for those over 55 years. Younger participants aged 18 to 25 years were more likely to use sunblock products than older participants (p = 0.001). Of those between 18 and 25 years old, 24.3% (n = 141) usually used sunblock, 22.2% (n = 129) sometimes used it, 36.9% (n = 214) never used it, and 16.6% (n = 96) rarely used it. For ages 26 to 35, this was 18.0% (n = 22) usually, 21.3% (n = 26) sometimes, 45.9% (n = 56) never, and 14.8% (n = 18) rarely. Dietary intake of foods containing vitamin D, zinc, and ferritin was comparable across age groups (p = 0.471). However, supplement use was significantly higher in older participants aged above 45 years compared to younger participants (p = 0.001). Of those aged between 18 and 25, 30.5% (n = 177) took supplements, while 69.5% (n = 403) did not. In those aged between 26 and 35, 41.8% (n = 51) were taking supplements, while 58.2% (n = 71) were not.

Physical activity levels were similar across age groups, with around half of all participants reporting average activity levels and no significant differences observed (p = 0.566). Daily sun exposure and duration of exposure also did not differ significantly by age (p = 0.186 and p = 0.069, respectively). However, younger participants aged 18 to 25 years were more likely to use sunblock products than older participants (p = 0.001). Dietary intake of foods containing vitamin D, zinc, and ferritin was comparable across age groups (p = 0.471). In contrast, supplement use was significantly higher in older participants aged above 45 years compared to younger participants (p = 0.001).

Table 5 shows the differences in practices by gender. Male participants were likelier to report being physically active than females (p = 0.001). Of the males, 26.5% (n = 114) were less active, 50.8% (n = 219) had average activity, and 22.7% (n = 98) were more active. In females, 29.2% (n = 162) were less active, 58.7% (n = 325) had average activity, and 12.1% (n = 67) were more active. Males also had significantly higher sun exposure, with more spending over 20 minutes daily in the sun (p = 0.001). Of the males, 58.9% (n = 254) had daily sun exposure, 10.7% (n = 46) did not, and 30.4% (n = 131) had it sometimes. In females, 16.2% (n = 90) had daily sun exposure, 29.4% (n = 163) did not have any, and 54.3% (n = 301) only had it sometimes.

**Table 5:** Assessment of differences in practices with regard to gender P: Pearson's chi-squared test X2, *p < 0.05 (significant)

Questions on practices	Category	Gender		P-value
		Male, N (%)	Female, N (%)	
Compared to your peers of the same age, do you think you are more active, less active, or average?	Less active	114 (26.5%)	162 (29.2%)	0.001*
	Averagely active	219 (50.8%)	325 (58.7%)	
	More active	98 (22.7%)	67 (12.1%)	
Are you exposed to sunlight daily?	Yes	254 (58.9%)	90 (16.2%)	0.001*
	No	46 (10.7%)	163 (29.4%)	
	Sometimes	131 (30.4%)	301 (54.3%)	
What is the duration of your daily sun exposure?	Less than 20 minutes	223 (51.7%)	482 (87.0%)	0.001*
	More than 20 minutes	208 (48.3%)	72 (13.0%)	
Do you use sunblock products?	Usually	22 (5.1%)	159 (28.7%)	0.001*
	Sometimes	43 (10.0%)	192 (34.7%)	
	Never	306 (71.0%)	103 (18.6%)	
	Rarely	60 (13.9%)	100 (18.1%)	
Do you usually eat foods that contain vitamin D, zinc, and ferritin (fish, egg yolks, milk, cheese, yogurt)?	Usually	258 (59.9%)	268 (48.4%)	0.002*
	Sometimes	148 (34.3%)	244 (44.0%)	
	Rarely	25 (5.8%)	42 (7.6%)	
Do you take vitamin D, zinc, and ferritin supplements?	Yes	121 (28.1%)	228 (41.2%)	0.001*
	No	310 (71.9%)	326 (58.8%)	

Of the males, 51.7% (n = 223) had less than 20 minutes of daily sun exposure, and 48.3% (n = 208) had over 20 minutes. In females, 87.0% (n = 482) had less than 20 minutes, and 13.0% (n = 72) had over 20 minutes. Females were usually more likely to use sunblock products than males (p = 0.001). Of the males, 5.1% (n = 22) usually used sunblock, 10.0% (n = 43) sometimes used it, 71.0% (n = 306) never used it, and 13.9% (n = 60) rarely used it. For females, this was 28.7% (n = 159) usually, 34.7% (n = 192) sometimes, 18.6% (n = 103) never, and 18.1% (n = 100) rarely.

Females were also more likely to eat foods containing vitamin D, zinc, and ferritin (p = 0.002). Of the males, 59.9% (n = 258) usually ate these foods, 34.3% (n = 148) sometimes ate them, and 5.8% (n = 25) rarely ate them. For females, this was 48.4% (n = 268) usually, 44.0% (n = 244) sometimes, and 7.6% (n = 42) rarely. Supplement use was significantly higher among females than males (p = 0.001). Of the males, 28.1% (n = 121) took supplements, while 71.9% (n = 310) did not. In females, 41.2% (n = 228) took supplements, and 58.8% (n = 326) did not. Male participants were likelier to report being physically active than females (p = 0.001). Males also had significantly higher sun exposure, with over 20 minutes daily in the sun (p = 0.001). Females were usually more likely to use sunblock products than males (p = 0.001). They were also more likely to eat foods containing vitamin D, zinc, and ferritin (p = 0.002). Supplement use was significantly higher among females than males (p = 0.001).

Table 6 explores the association between hair loss and sociodemographic factors. Overall, most participants in all sociodemographic groups believed there was a relationship between these nutritional deficiencies and hair loss, with yes responses ranging from 55.6% to 100%.

**Table 6:** Assessment of the association between hair loss and sociodemographic factors P: Pearson's chi-squared test X2, *p < 0.05 (significant)

Variable	Category	Is there a relationship between vitamin D, zinc, ferritin deficiency, and hair loss?			P-value
		Yes, N (%)	No, N (%)	I don't know, N (%)	
Age	26 to 35	88 (72.1)	6 (4.9)	28 (23.0)	0.274
	36 to 45	131 (77.5)	1 (0.6)	37 (21.9)	
	18 to 25	420 (72.4)	13 (2.2)	147 (25.3)	
	46 to 55	79 (78.2)	1 (1.0)	21 (20.8)	
	>55	11 (84.6)	0 (0.0)	2 (15.4)	
Gender	Male	271 (62.9)	14 (3.2)	146 (33.9)	0.001*
	Female	458 (82.7)	7 (1.3)	89 (16.1)	
Marital status	Widower	4 (80.0)	0 (0.0)	1 (20.0)	0.316
	Single	464 (72.3)	17 (2.6)	161 (25.1)	
	Married	251 (76.5)	4 (1.2)	73 (22.3)	
	Divorce	10 (100.0)	0 (0.0)	0 (0.0)	
Nationality	Saudi	719 (74.4)	17 (1.8)	231 (23.9)	0.001*
	Non-Saudi	10 (55.6)	4 (22.2)	4 (22.2)	
Living	City	294 (73.0)	12 (3.0)	97 (24.1)	0.301
	Village	435 (74.7)	9 (1.5)	138 (23.7)	
Knowledge level	Low	308 (71.5)	9 (2.1)	114 (26.5)	0.242
	High	421 (76.0)	12 (2.2)	121 (21.8)	

Of those aged between 26 and 35 years, 72.1% (n = 88) responded yes, 4.9% (n = 6) responded no, and 23.0% (n = 28) responded I don't know. For ages 36 to 45, 77.5% (n = 131) responded yes, 0.6% (n = 1) responded no, and 21.9% (n = 37) responded I don't know. Among ages 18 and 25, 72.4% (n = 420) responded yes, 2.2% (n = 13) responded no, and 25.3% (n = 147) responded I don't know. For ages 46 to 55, 78.2% (n = 79) responded yes, 1.0% (n = 1) responded no, and 20.8% (n = 21) responded I don't know. Of those over 55 years old, 84.6% (n = 11) responded yes, and 15.4% (n = 2) responded I don't know.

A higher percentage of females (82.7%, n = 458) responded yes than males (62.9%, n = 271). Of the males, 3.2% (n = 14) responded no, and 33.9% (n = 146) responded I don't know. In females, 1.3% (n = 7) responded no, and 16.1% (n = 89) responded I don't know. Among the Saudis, 74.4% (n = 719) responded yes, 1.8% (n = 17) responded no, and 23.9% (n = 231) responded I don't know. As for non-Saudis, 55.6% (n = 10) responded yes, 22.2% (n = 4) responded no, and 22.2% (n = 4) responded I don't know. The p-values indicate statistically significant differences based on gender (p = 0.001) and nationality (p = 0.001) but there were no significant differences based on age, marital status, living area, or knowledge level.

Table 7 explores the association between hair loss and various health practices. Of those less active, 69.9% (n = 193) responded yes, 4.0% (n = 11) responded no, and 26.1% (n = 72) responded I don't know. For those with average activity, 74.1% (n = 403) responded yes, 0.9% (n = 5) responded no, and 25.0% (n = 136) responded I don't know. Of those more active, 80.6% (n = 133) responded yes, 3.0% (n = 5) responded no, and 16.4% (n = 27) responded I don't know. Of those with daily sun exposure, 70.6% (n = 243) responded yes, 3.5% (n = 12) responded no, and 25.9% (n = 89) responded I don't know. For those without daily exposure, 75.6% (n = 158) responded yes, 2.4% (n = 5) responded no, and 22.0% (n = 46) responded I don't know. Of those with sometimes sun exposure, 75.9% (n = 328) responded yes, 0.9% (n = 4) responded no, and 23.1% (n = 100) responded I don't know.

**Table 7:** Assessment of the association between hair loss and practices P: Pearson's chi-squared test X2, *p < 0.05 (significant)

Variables	Category	Is there a relationship between vitamin D, zinc, ferritin deficiency, and hair loss?			P-value
		Yes, N (%)	No, N (%)	Don't know, N (%)	
Compared to your peers of the same age, do you think you are more active, less active, or average?	Less active	193 (69.9)	11 (4.0)	72 (26.1)	0.004*
	Averagely active	403 (74.1)	5 (0.9)	136 (25.0)	
	More active	133 (80.6)	5 (3.0)	27 (16.4%)	
Are you exposed to sunlight daily?	Yes	243 (70.6)	12 (3.5)	89 (25.9)	0.100
	No	158 (75.6)	5 (2.4)	46 (22.0)	
	Sometimes	328 (75.9)	4 (0.9)	100 (23.1)	
What is the duration of your daily sun exposure?	Less than 20 minutes	536 (76.0)	10 (1.4)	159 (22.6)	0.011*
	More than 20 minutes	193 (68.9)	11 (3.9)	76 (27.1)	
Do you use sunblock products?	Usually	147 (81.2)	4 (2.2)	30 (16.6)	0.001*
	Sometimes	199 (84.7)	5 (2.1)	31 (13.2)	
	Never	266 (65.0)	8 (2.0)	135 (33.0)	
	Rarely	117 (73.1)	4 (2.5)	39 (24.4)	
Do you usually eat foods that contain Vitamin D, Zinc, and Ferritin (fish, egg yolks, milk, cheese, yogurt)?	Usually	405 (77.0)	8 (1.5)	113 (21.5)	0.124
	Sometimes	279 (71.2)	10 (2.6)	103 (26.3)	
	Rarely	45 (67.2)	3 (4.5)	19 (28.4)	
Do you take vitamin D, zinc, and ferritin supplements?	Yes	291 (83.4)	12 (3.4)	46 (13.2)	0.001*
	No	438 (68.9)	9 (1.4)	189 (29.7)	

Of those with <20 minutes of sun exposure, 76.0% (n = 536) responded yes, 1.4% (n = 10) responded no, and 22.6% (n = 159) responded I don't know. For those with >20 minutes of sun exposure, 68.9% (n = 193) responded yes, 3.9% (n = 11) responded no, and 27.1% (n = 76) responded I don't know. Of those who usually use sunblock, 81.2% (n = 147) responded yes, 2.2% (n = 4) responded no, and 16.6% (n = 30) responded I don't know. For those who sometimes use it, 84.7% (n = 199) responded yes, 2.1% (n = 5) responded no, and 13.2% (n = 31) responded I don't know. Of supplement users, 83.4% (n = 291) responded yes, 3.4% (n = 12) responded no, and 13.2% (n = 46) responded I don't know. For non-users, 68.9% (n = 438) responded yes, 1.4% (n = 9) responded no, and 29.7% (n = 189) responded I don't know.

Those with shorter daily sun exposure (<20 minutes) were more likely to answer yes (536, 76.0%) versus those with longer exposure (193, 68.9%) (p = 0.011). People who usually or sometimes use sunblock believed in the relationship significantly more than those who rarely or never use it (81.2% to 84.7% vs. 65.0% to 73.1%) (p = 0.001). Finally, those who take vitamin D, zinc, and ferritin supplements were more likely to answer yes (291, 83.4%) compared to non-users (438, 68.9%) (p = 0.001). In contrast, daily sun exposure and dietary intake of vitamin D and mineral-rich foods had no statistically significant association. Still, the majority in all practice categories believed the relationship existed.

The regression analysis between hair loss and various factors in Table 8 revealed several significant and non-significant relationships. An examination of the different age groups showed that age was not significantly related to hair loss. For the age groups 26 to 35, 36 to 45, and 46 to 55, the p-values were 0.903, 0.532, and 0.552, respectively, indicating the lack of a significant relationship. Even in the age group above 55, the relationship was insignificant (p = 0.192). However, gender showed a significant association with hair loss. Female gender was significantly associated with hair loss (B = -0.344, p < 0.0001), indicating that being female was associated with a decrease in hair loss compared to males.

**Table 8:** Regression analysis between hair loss and other factors

Variable	B	Standard error	Beta	t	Significant	95% CI lower	95% CI upper
Age (26 to 35)	0.010	0.082	0.004	0.121	0.903	-0.151	0.171
Age (36 to 45)	-0.045	0.072	-0.020	-0.625	0.532	-0.187	0.097
Age (46 to 55)	-0.053	0.090	-0.019	-0.594	0.552	-0.230	0.123
Age (>55)	-0.302	0.232	-0.040	-1.306	0.192	-0.757	0.152
Gender (female)	-0.344	0.059	-0.200	-5.786	0.000	-0.461	-0.227
Total knowledge (low)	0.064	0.053	0.037	1.199	0.231	-0.041	0.168
Residence (village)	-0.006	0.054	-0.003	-0.103	0.918	-0.111	0.100
Heard about vitamins (no)	0.475	0.125	0.119	3.797	0.000	0.229	0.720
Exposed to sunlight (no)	0.026	0.067	0.012	0.381	0.704	-0.107	0.158
Duration of sun exposure (<20 minutes)	0.045	0.064	0.024	0.701	0.483	-0.081	0.172
Eat foods that contain vitamins (rarely)	0.108	0.105	0.032	1.028	0.304	-0.098	0.313
Take vitamins (no)	0.241	0.056	0.135	4.277	0.000	0.130	0.351

Total knowledge, classified as low or high, showed no significant relationship with hair loss (p = 0.231). Similarly, residency in a village or a city was not significantly related to hair loss (p = 0.918). A lack of awareness about vitamins showed a significant positive relationship with hair loss (B = 0.475, p < 0.0001). This indicates that individuals who had not heard about vitamins were more likely to experience hair loss. However, lack of exposure to sunlight did not significantly affect hair loss (p = 0.704), and the duration of sun exposure (<20 minutes) also showed no significant relationship with hair loss (p = 0.483).

Eating foods rarely containing vitamins did not significantly relate to hair loss (p = 0.304). However, not taking vitamin supplements showed a significant positive relationship with hair loss (B = 0.241, p < 0.0001), indicating that individuals who did not take vitamin supplements were more likely to experience hair loss. The regression analysis showed that gender, awareness about vitamins, and vitamin supplement intake were significant factors related to hair loss.

## Discussion

This study evaluated the relationship between hair loss and various factors, including vitamin D, zinc, and ferritin deficiencies, the knowledge and practices concerning these deficiencies, and different sociodemographic characteristics. The results revealed several significant findings that align with existing literature, expanding our understanding of hair loss's multifactorial nature. Most participants were young adults aged between 18 and 25, single, and female. This was similar to past research showing that young people, especially those who are single, are generally more willing to participate in health surveys, and women tend to have higher response rates [12-15].

Most participants had heard about vitamin D, zinc, and ferritin and identified sunlight as a source of these vitamins and minerals, consistent with past studies [4,12,16-20]. According to research conducted in Al-Qunfudhah, Saudi Arabia, even though 91% of participants had previously heard about vitamin D, only 17.4% could identify sunlight exposure as a primary source of vitamin D [19]. Previous research revealed the importance of sunlight in vitamin D synthesis [21,22]. However, only 33.4% knew about cardiovascular benefits, lower than in past reports. In the current study, nearly half have high knowledge, whereas 431 (43.8%) have low knowledge about vitamin D, zinc, and ferritin deficiencies and the associated risk of hair loss. This aligns with past studies that revealed insufficient knowledge [4,12,18-20,23]. Saudi research in Riyadh found poor knowledge and inadequate practice [24]. They were highlighting a continued knowledge gap. Lack of sun exposure and excessive sunscreen use were correctly identified as the top causes of deficiency. Moreover, a lack of awareness about vitamins was significantly correlated with increased hair loss, emphasizing the critical role of knowledge and awareness in managing health problems such as hair loss. These findings echo previous research that underscored the significance of awareness and understanding of health issues for effective prevention and management [12,25,26].

The study found significant gender differences in several aspects. Females were associated with a decrease in hair loss compared to males, which could be related to hormonal differences that affect hair growth and loss [12,27]. Additionally, women were more inclined to accept the link between deficiencies in vitamin D, zinc, ferritin, and hair loss. This could be attributed to a higher health consciousness in females than males [12,26,28].

Regarding practices, only 34.9% (n = 344) had daily sun exposure, with 705 (71.6%) having sun exposure for under 20 minutes, similar to earlier research showing low sun exposure [12,29,30]. Previous studies in Saudi Arabia found that sun exposure was limited due to extreme heat, societal beliefs about covering the body, and a lack of infrastructure that makes sun exposure difficult [31]. Previous studies showed that females reported higher supplement use, food intake, and sunblock use [12,32]. In an earlier study, 46.5% of females were exposed to sunshine, and the majority of 51.2% revealed themselves for 10 to 29 minutes, which is a more significant percentage than ours, who reported applying sunscreen (43.9%) [30].

Participants who reported being physically active were more likely to believe in the relationship between vitamin D, zinc, ferritin deficiencies, and hair loss. This finding aligns with studies indicating that individuals engaging in regular physical activity are often more knowledgeable about health-related issues [33]. Moreover, participants with less sun exposure and those using sunblock products were likelier to believe in the relationship. This can be explained by the understanding that sunlight exposure is necessary for vitamin D synthesis, and sunblock overuse could cause vitamin deficiency [34].

Not taking vitamin supplements showed a significant positive relationship with hair loss, underlying the importance of vitamin supplement intake. This aligns with several studies that indicated nutrient deficiencies, such as iron, vitamin D, and zinc, can contribute to hair loss, and supplementation may aid in restoring hair health [3,4,12]. In the research conducted in Majmaah, Saudi Arabia, 17% said there was a link between vitamin D insufficiency and hair loss [4].

In our study, the Internet was the top source of information (37.1%, n = 365), reflecting the increasing reliance on web-based health resources seen in prior research [35]. A previous study in Saudi Arabia found that the media (27.9%) was the most prevalent source of information, followed by healthcare professionals and family and friends (21.4%) [4]. In another study in Saudi Arabia, the doctor was the leading source of information (37.4%), followed by TV shows (34.8%) and the Internet (32.3%), while friends represented 18.7% [30]. According to research conducted in Jeddah, Saudi Arabia, the largest source of knowledge was friends (75.8%), followed by family (70.6%), physicians (48.1%), and the media (35.8%) [20]. It is essential to encourage the population to use the correct sources of health information.

The findings from this study are consistent with previous research and provide new insights into the complex relationship between hair loss, vitamin deficiencies, and individual knowledge and lifestyle practices. The significant findings emphasize the importance of knowledge and awareness about vitamins and their deficiencies, gender differences, and the role of lifestyle factors in hair loss. It also highlights the need for further research to better understand and manage hair loss.

Study limitations

The participant group is predominantly composed of young, single Saudi citizens, yet the demographic skewness could limit the applicability of our findings to other age groups, marital statuses, or nationalities. The study's cross-sectional design prevents us from establishing causality between the investigated variables, necessitating longitudinal studies for a more effective cause-and-effect assessment. Furthermore, most participants identified the Internet as their principal source of information about deficiencies, a source with varying degrees of accuracy and reliability. Lastly, the study's scope, which is primarily on vitamin D, zinc, and ferritin deficiencies, doesn't address other potential nutrients impacting hair health and loss, thus pointing to an area for further exploration.

## Conclusions

The survey results indicate that most participants are aware of vitamin D, zinc, and ferritin deficiencies, with most recognizing sunlight as a source of these essential nutrients. The Internet and medical professionals were the primary sources of information, underscoring the vital role of these platforms in disseminating health-related knowledge. There is a common understanding among participants that deficiencies in these nutrients are related to hair loss. Although widespread, this belief is more prevalent among females and Saudi nationals.

Practices such as physical activity, use of sunblock, and taking supplements, among others, were significantly related to the belief in the association between these deficiencies and hair loss. Moreover, gender, awareness of vitamins, and intake of vitamin supplements appear to be significant factors concerning hair loss. The results highlight the importance of awareness and knowledge about nutritional deficiencies and their potential health implications. However, despite the high levels of awareness, the practice of preventive measures, like sufficient sun exposure and dietary intake of nutrient-rich foods, remains subpar.

The lack of significant differences between age groups in practices such as sun exposure and dietary habits suggests that more targeted interventions may be necessary to promote these preventive measures across all age groups. The findings underscore the need for continued public health education and interventions to enhance knowledge and promote healthy practices to prevent these deficiencies.
